# Supplementary material for: High-Fat Diet Impairs Muscle Function and Increases the Risk of Environmental Heatstroke in Mice
Source: Int J Mol Sci. 2022 May 9;23(9):5286. doi: 10.3390/ijms23095286 (PMC9104075; doi:10.3390/ijms23095286)
Supplement: Supplementary file 1 [file ijms-23-05286-s001.zip › ijms-1684357-supplementary.pdf]

SUPPLEMENTAL MATERIAL

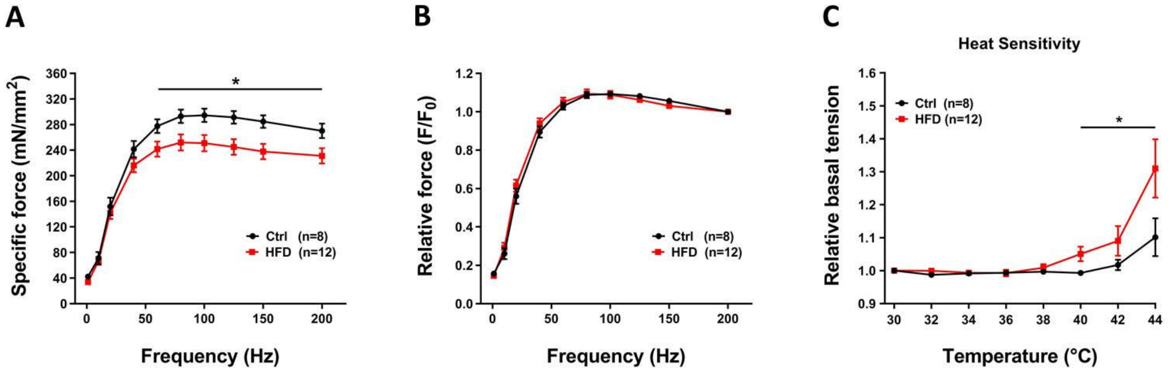

Figure S1. In-vitro specific force and temperature dependence of basal tension in Soleus muscles. (**A** and **B**) Force frequency (1-200Hz) curve of specific force (panel **A**) and relative force normalized to 200Hz (panel **B**) in isolated Soleus muscles. (**C**) Relative basal tension (normalized to 30°C) during exposure to increasing temperature (from 30 to 44ºC). Data are shown as mean ± SEM (*p<0.05), as evaluated two-way ANOVA followed by Tukey´s post-hoc test. n = number of Soleus muscles tested.

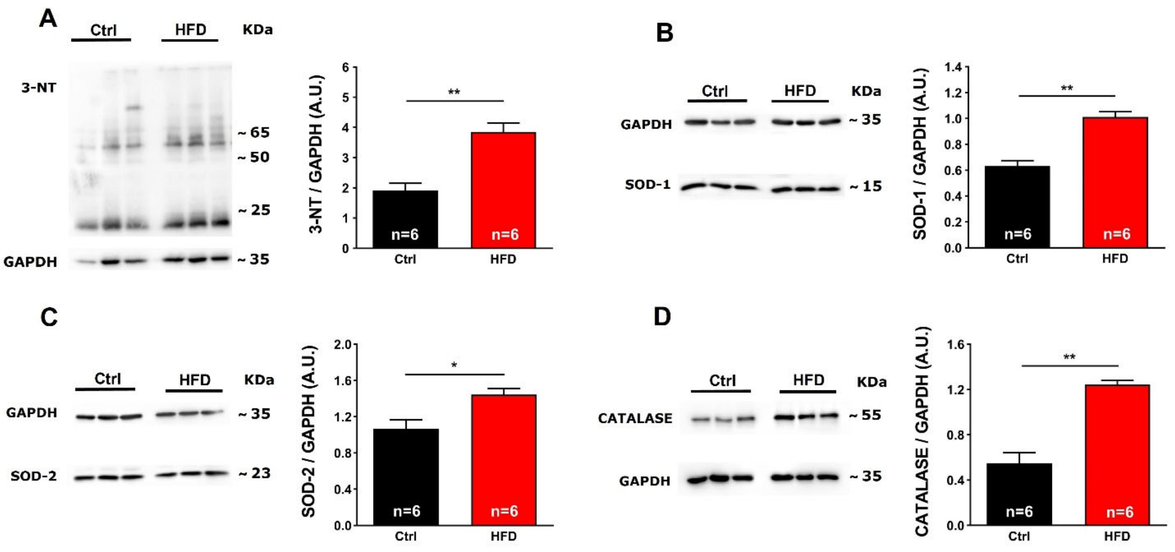

Figure S2. Markers of oxidative and nitrosilative stress in Soleus muscles. Representative immunoblots (left) and relative band densities normalized to GAPDH levels (right) of 3-NT (panel **A**), SOD-1 (panel **B**), SOD-2 (panel **C**), and Catalase (panel **D**) in Soleus muscle homogenates. Data are shown as mean ± SEM (*p<0.05 and **p<0.01), as evaluated by two-tailed unpaired Student's t-test. n = number of mice tested.

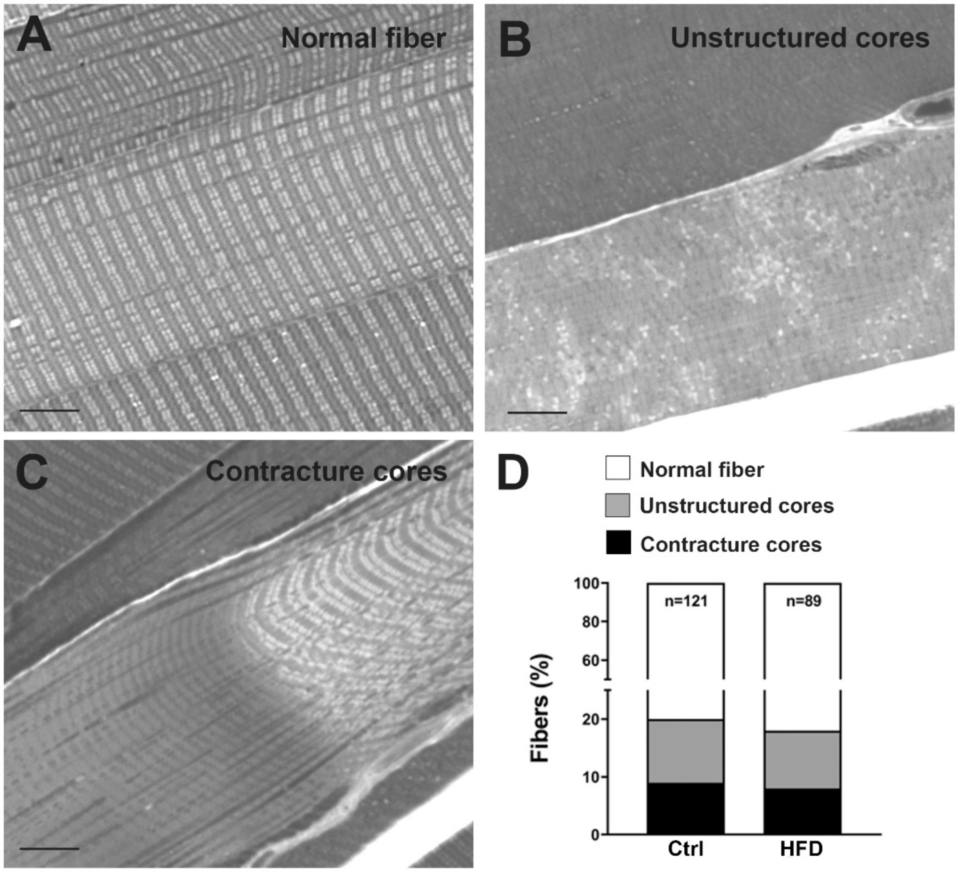

Figure S3. Muscle damage after the heat stress protocol evaluated by histological analysis. Quantitative analysis (panel **D**) in histological sections of EDL fibers, which were classified in three groups: presenting normal appearance (as in panel **A**), containing areas with loos of striation (as in panel **B**), or finally containing contracture cores (as in panel **C**). n = number of EDL fibers analyzed.

Table S1. Energetic and nutrients composition of the diets administrated to Ctrl and HFD mice.

(Ctrl Diet)

Metabolized energy

Content	Value (Kcal/Kg)	Percentage (%)
Fat	3.780	70
Protein	842	16
Carbonhydrates	766	14

Crude nutrients and moisture

Content	Value (mg/Kg)	Percentage (%)
Moisture	44.328	4.4
Crude Ash	61.481	6.1
Crude Fibre	47.350	4.7
Crude Fat	420.018	42.0
Crude Protein	210.545	21.1
Nitrogenfree Extractives	216.278	21.7

(HF Diet)

Metabolized energy

Content	Value (Kcal/Kg)	Percentage (%)
Fat	358	10
Protein	828	24
Carbonhydrates	2.329	66

Crude nutrients and moisture

Content	Value (mg/Kg)	Percentage (%)
Moisture	79.090	7.9
Crude Ash	43274	4.3
Crude Fibre	30.829	3.1
Crude Fat	39.725	4.0
Crude Protein	206.900	20.7
Nitrogenfree Extractives	600.183	60

Table S2. Manufacturer and catalogue number by material reagent used.

CHEMICAL/REAGENT	MANUFACTURER	CATALOGUE NUMBER
**2-mercaptoethanol**	Sigma-Aldrich, USA	M3148
**Anti-3-NT primary antibody**	Merck Millipore, Italy	05-233
**Anti-Catalase primary antibody**	Santa Cruz Biotechnology Inc., USA	SC-271803
**Anti-GAPDH primary antibody**	OriGene Technologies Inc., USA	TA802519
**Anti-SOD1 primary antibody**	Santa Cruz Biotechnology Inc., USA	SC-11407
**Anti-SOD2 primary antibody**	Santa Cruz Biotechnology, Inc., USA	SC-30080
**BCA quantification kit**	ThermoFisher scientific, USA	23225
**$CaCl_2$**	Sigma-Aldrich, USA	C8106
**Caffeine**	Sigma-Aldrich, USA	C0750
**Calcium colorimetric assay kit**	Sigma-Aldrich, USA	MAK022
**Creatine Kinase colorimetric assay kit**	Sigma-Aldrich, USA	MAK116-1KT
**DPX mounting medium for histology**	Sigma-Aldrich, USA	06522
**EDTA**	Sigma-Aldrich, USA	ED2SS
**Enhanced chemiluminescent liquid**	Perkin-Elmer, USA	NEL104001EA
**Epoxy resin (Epon 812)**	Electron Microscopy Sciences, USA	14120
**Glucose**	Sigma-Aldrich, USA	G8270
**Glutaraldehyde**	Histo-line Laboratories, Italy	18428-10
**HEPES**	Sigma-Aldrich, USA	H3375
**Horseradish peroxidase conjugated secondary antibodies (anti-mouse)**	Merck Millipore, USA	401253
**Horseradish peroxidase conjugated secondary antibodies (anti-rabbit)**	Merck Millipore, USA	401393
**KCl**	Sigma-Aldrich, USA	P9333
**$MgSO_4$**	Sigma-Aldrich, USA	M7506
**NaCaCO**	Electron microscopy sciences, USA	12300
**NaCl**	Sigma-Aldrich, USA	S7653
**NaH_2PO_4**	Sigma-Aldrich, USA	71505
**Non-fat dry milk**	EuroClone, Italy	APA08300500
**OsO4**	Electron microscopy sciences, USA	19151
**Potassium turbidimetric assay kit**	MyBioSource, USA	MBS2540590
**Protease inhibitors cocktail**	Roche, Germany	11836153001
**SDS**	Bio-Rad, USA	1610301
**Sodium Borate Tetra**	Electron microscopy sciences, USA	21130
**Sodium Deoxycholate**	Sigma-Aldrich, USA	D6750
**Toluidine blue O**	Electron microscopy sciences, USA	22050
**TRIS HCl**	Sigma-Aldrich, USA	T3253
**Triton-X**	Sigma-Aldrich, USA	T8787
**Tween 20**	Sigma-Aldrich, USA	P1379
